# Thoracoscopic removal of impacted denture: Report of a case with review of literature

**DOI:** 10.4103/0972-9941.72600

**Published:** 2010

**Authors:** Abhay N Dalvi, Vinay K Thapar, Sachin Jagtap, Devyani J Barve, Dattaraj P Savarkar, Mahadev N Garle, Akash P Shukla

**Affiliations:** Department of Surgery, Seth G S Medical College and King Edward VII Memorial Hospital, Mumbai, India; 1 Department of Gastroenterology, Seth G S Medical College and King Edward VII Memorial Hospital, Mumbai, India

**Keywords:** Dentures, oesophageal foreign body, thoracoscopy

## Abstract

Impacted foreign bodies in the oesophagus are common. Because of their large size, rigidity and pointed edges, dentures get frequently impacted in the oesophagus and are difficult for endoscopic retrieval. Traditional thoracotomy for retrieval of impacted foreign bodies in the thoracic oesophagus is associated with significant morbidity. We present a case of impacted denture in the mid-oesophagus successfully removed using minimal access thoracoscopic procedure.

## INTRODUCTION

Accidental foreign body (FB) ingestion is a common problem in clinical practice. Once an FB has been swallowed beyond the cricopharyngeus, it frequently remains in the oesophagus as it has weak peristalsis and multiple anatomical narrowings.[1] Dentures are common accidentally ingested foreign bodies, especially in the elderly population.[2] Because of their large size and pointed edges, they get frequently impacted and associated with high morbidity and mortality.[2] Longstanding impaction can lead to ulceration, inflammation, perforation and sepsis leading to death.[3] Endoscopic removal of impacted dentures can cause oesophageal tears and is therefore dangerous. Surgery is required for retrieval of the impacted denture.[4] We present a case of a denture impacted in the oesophagus successfully managed by thoracoscopy.

## CASE REPORT

**Figure 1:**
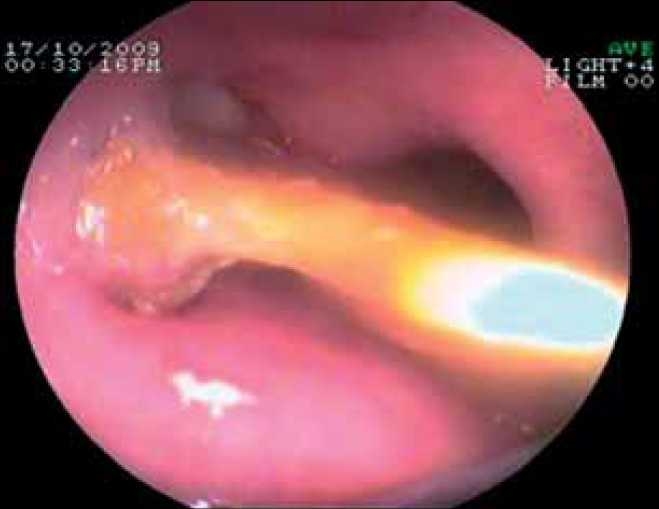
Endoscopic view of impacted denture.

A 65-year-old woman presented to a primary health care centre with dysphagia, odynophagia and a missing denture. She was treated symptomatically hoping that the denture will pass on its own and referred to our institute 30 days after ingestion. At presentation her vitals were stable. X-rays of the neck and chest were normal. Flexible upper GI endoscopy revealed impacted denture in the mid-oesophagus at 29 cm with one of its flanges embedded in the oesophageal wall [Figure 1]. Computerised tomography (CT) scan of the chest revealed transversely stretched segment of the mid-oesophagus with suspected partial penetration of the wall.

We planned endoscopic removal of the denture with surgical extraction in the same setting if the endoscopy failed under anaesthesia. However, as part of the denture was impacted in the wall, we abandoned the endoscopy to avoid oesophageal tear.

**Figure 2:**
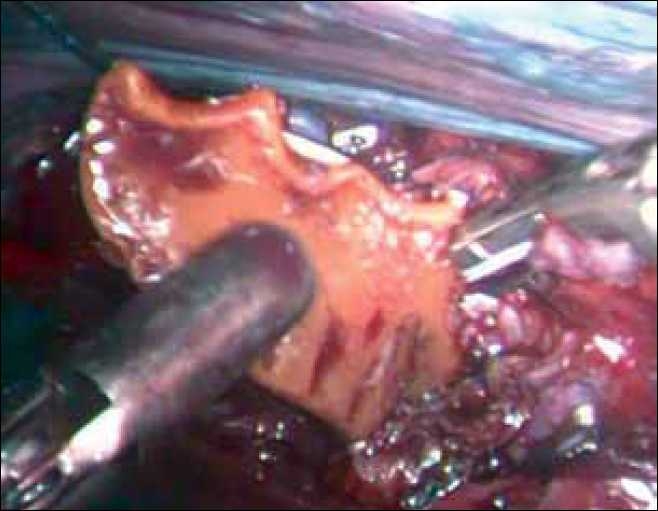
The denture being extracted. Note the ligature and clip on the azygous vein.

The patient was placed in 90° left lateral position. Four ports were used: 10-mm optical port in the 5^th^ intercostal space (ICS) in the posterior axillary line, 5-mm port in the 4^th^ ICS in the mid-axillary line, 10-mm port in the 7^th^ ICS in the mid-axillary line and 5-mm port in the 7^th^ ICS in the anterior axillary line for the fan-shaped retractor to retract the lung anteriorly. The denture was located just behind and above the azygous vein. The azygous vein was ligated and divided. The pleura was incised over the oesophagus, and a 5-cm segment of the involved oesophagus was freed. A small incision was made on the oesophagus at the level of impacted denture, which was removed with blunt forceps [Figure 2]. The defect in the oesophagus was closed with interrupted 3-0 mersilk sutures. The ryles tube was positioned in the oesophagus with its holes at the level of the perforation. The denture was removed through one of the ports after dividing it into two pieces. A 24-french intercostal drain was placed through one port, and the ports were closed. A feeding jejunostomy was done. The patient had a minor localised leak postoperatively, which settled with conservative management.

## DISCUSSION

Dentures, apart from improving facial aesthetics, make eating a more pleasant experience and enhance clarity of speech. With an increase in the denture-wearing population, there is an increase in the incidence of impacted dentures.[4] It has been suggested that wearing dentures decreases the sensation in the oral cavity, thereby increasing the incidence of FB ingestion. Impacted dentures can cause mucosal ulceration, inflammation and fatal complications such as perforation, para- or retro-oesophageal abscess, mediastinitis, empyema or even tracheo and aorto-oesophageal fistula.[2]

Following FB ingestion, patients usually present with dysphagia and odynophagia. Other symptoms are hypersalivation, retrosternal fullness and regurgitation of food. The diagnosis is usually easily elicited if the patient is able to give a reliable history. Radiology can determine the exact site of radiopaque impacted FB and air entrapment if there is perforation. Dentures, however, are frequently made of radiolucent acrylic resin, though the radiopaque wire clasps of the denture can sometimes be seen.[4] In our patient, x-ray failed to reveal the denture as it was retrocardiac and obscured by the cardiac shadow. However, CT showed a dilated transversely stretched oesophagus with possible wall penetration by the denture.

Endoscopic extraction of dentures carries a high risk of perforation (23%).[5] In addition, periesophagitis at the site of impaction increases the risk of perforation. In our patient as the denture was impacted for more than a month, endoscopic extraction was impossible due to partial migration into the wall.

Open oesophagotomy has been suggested as the safest and the most effective method of removing impacted dentures.[5] Cervical oesophagotomy for impacted upper oesophageal dentures has low morbidity. However, thoracotomy for removal of FB in the thoracic oesophagus is associated with high morbidity as most of these patients are old and have coexisting illness.[1,2] Thoracoscopic approach can avoid all the morbidity of open thoracotomy.

We preferred the right thoracoscopic approach as the mid-oesophagus on the left side is obscured by the aortic arch and the heart. Lateral position was preferred for ease of conversion to open thoracotomy in case of difficulty. Though thoracoscopic removal of foreign bodies that have migrated from the oesophagus to the mediastinum is reported,[6,7] impacted denture involves the problem of locating the denture, opening up the oesophagus and suturing it. As the denture was situated in the oesophageal segment near the azygous vein, we had to divide the vein in order to expose the segment of the oesophagus. Experience with endoscopic suturing allowed us to securely close the oesophagotomy with interrupted sutures. Review of literature reveals only one previous successful thoracoscopic removal of denture.[8]

## CONCLUSION

With increasing aging of the population, the incidence of dental prostheses occurring as oesophageal foreign bodies will increase in the future. It is therefore important to educate elderly patients about the dangers of accidental swallowing of dental prostheses and to pay attention to the stability of dental prostheses inside the oral cavity. Endoscopic extraction of impacted dentures is associated with a high risk of perforation, and surgery is the treatment of choice for impacted longstanding dentures. Thoracoscopy minimises the trauma of thoracotomy and has a definite role to play in the retrieval of impacted oesophageal foreign bodies.
